# Recombinant Fibrous Protein Gels as Rheological Modifiers in Skin Ointments

**DOI:** 10.1021/acsapm.4c02468

**Published:** 2024-10-08

**Authors:** Dustin Britton, Jonathan Sun, Hammad Ali Faizi, Ligeng Yin, Wei Gao, Jin Kim Montclare

**Affiliations:** † Department of Chemical and Biomolecular Engineering, New York University Tandon School of Engineering, Brooklyn, New York 11201, United States; ‡ Department of Chemistry, New York University, New York, New York 10012, United States; § The Dow Chemical Company, Home and Personal Care, Midland, Michigan 48611, United States; ▽ The Dow Chemical Company, Home and Personal Care, Collegeville, Pennsylvania 19426, United States; ¶ The Dow Chemical Company, Analytical Science, Collegeville, Pennsylvania 19426, United States; & Bernard and Irene Schwartz Center for Biomedical Imaging, Department of Radiology, New York University School of Medicine, New York, New York 10016, United States; $ Department of Biomaterials, New York University College of Dentistry, New York, New York 10010, United States; @ Department of Biomedical Engineering, New York University, New York, New York 11201, United States

**Keywords:** coiled-coil protein, rheological modifier, pH-responsive material, emulsion, hydrogel

## Abstract

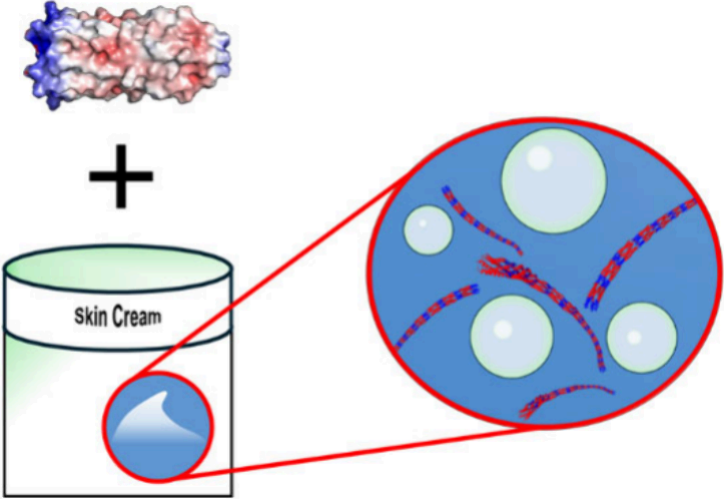

Rheological modifiers are an important component in the development of skin cream (SC) chassis for personal skin care products (PSCPs). The viscous behavior of a PSCP is critical to its effectiveness where its uniformity and material strength impact its processing, storage, and delivery of active ingredients. Due to the mildly acidic environment of the skin, PSCPs require a SC that will assist in maintaining their material strength at low pHs. We have investigated a coiled-coil protein hydrogel system for the ability to possess pH-responsiveness, where physical cross-linking and material strength is controlled by pH relative to the isoelectric point (pI) of the protein. We recently designed a coiled-coil protein hydrogel variant, Q5, which possesses a relatively low pI that we hypothesized to have improved supramolecular assembly into a hydrogel at acidic conditions. We demonstrate that Q5 can retain a partial solution-to-gel transition at pH 6.0 and acts as a soft hydrogel by rheology. We further tested Q5 to act as a rheological modifier in a standard SC at pH 6.0 and pH 8.0 to test conditions mediated by pH changes in the skin environment. Q5 reveals the ability to uniquely increase material strength at low pH in comparison to a standard rheological modifier like hydroxyethyl cellulose (HEC), suggesting modular protein-based coiled-coil rheological modifiers can be used in PSCPs.

## Introduction

Personal skin care products (PSCPs) cover a broad spectrum of consumer goods, ranging from cosmetics, which aim to enhance the aesthetic or sensory experience of healthy skin, to pharmaceuticals, designed to combat underlying skin conditions and diseases.^1^ PSCPs are composed of (i) active ingredients and (ii) a delivery chassis to facilitate product administration.^2,3^ The chassis used in a PSCP is often a complex colloidal mixture such as an emulsion or gel to accommodate multiple components in immiscible phases and imbue specific rheological behavior.^2,3^ The rheological properties of a chassis play a critical role in determining the performance of PSCPs and often must be tuned for specific environmental conditions such as pH.^4^ In order to adjust PSCPs to a desirable rheological profile at physiological conditions, rheological modifiers such as clays, polysaccharides, and polymers, are employed in the chassis.^4^

The identity of PSCP components, including rheological modifiers, has also become a growing consideration because of the recent emphasis on their ethical sourcing and environmental sustainability.^1^ This heightened scrutiny has fueled substantial market growth with an anticipated compound annual industrial growth rate (CAGR) of 8.64%, reaching $74.12 billion USD by 2027.^5−7^ While most natural products use polysaccharides derived from starch or cellulose,^4^ recombinant proteins mass-produced through bacterial or yeast fermentation may offer an innovative, scalable alternative that confers enhanced efficacy and greater consistency compared to traditional sources. Proteins also benefit from their amphiphilic nature allowing for increased moisture^8,9^ and the ability to bind a variety of molecules through hydrogen bonding, electrostatic interaction, and hydrophobicity. Therefore, proteins exhibit versatility as emulsifiers or gelling agents.

Another important consideration in the formulation of PSCP chassis is their pH-responsive stability due to their exposure to the mildly acidic microenvironment of skin, generally recognized to exist at pH of 5.4–5.9.^10^ The low pH environment may induce excessive swelling, destabilization of chains, or disruption of the matrix that can lead to colloidal instability and eventual phase separation, posing a challenge for PSCPs.^11,12^ Developing a versatile SC chassis with robust rheological properties at physiological pH as well as under mildly acidic conditions would accelerate the formulation stage of new PSCPs.

We have previously generated a library of α-helical coiled-coil proteins capable of self-assembling into nanofibers.^13−18^ We have demonstrated that these coiled-coil protein nanofibers can be manipulated by redistribution of their electrostatic potential energies resulting in differences between a positively charged patch at their N-terminus and a negatively charged patch at their C-terminus.^13,16^ Subsequently, we have demonstrated that some of these fibers can undergo thermoresponsive physical cross-linking to generate hydrogels with upper critical solution temperature (UCST) behavior which we have dubbed as Q hydrogels.^13−15,17^ Among the coiled-coil hydrogel sequences, variant Q5 undergoes a complete sol–gel transition within 24 h at 4 °C, possessing a critical gelation time, t_c_, of 11.5 h, a greater than 2-fold increase to previously reported coiled–coiled hydrogels.^13^

We have also recently studied the ability for our Q protein to act as pH-responsive hydrogels.^19,20^ Q can undergo faster gelation times and possess greater material strength at pHs closer to its isoelectric point (pI).^20^ While its pH-responsiveness allows for Q to possess candidacy as a stimuli-responsive chassis ingredient for PSCPs, Q possesses a pI of 10.3 and exhibits complete disassembly at low pH.^20^ Alternatively, Q5 possesses a pI of 8.0, which we hypothesize to allow for supramolecular assembly at low pHs necessary for a robust PSCP chassis. Herein, we investigate the ability of Q5 to act as a rheological modifier for skin ointments. To assess its candidacy to resist deformation under acidic conditions, Q5 is characterized for its pH-dependent structure and self-assembly at pH 6.0 and pH 8.0. Q5 is further assessed in a standard SC as a biocompatible and environmentally friendly rheological modifier.

## Materials and Methods

### Materials

Chemically competent M15MA *Escherichia coli* (*E. coli*) cells were a gift from David Tirrell (California Institute of Technology).^21^ Bacto-tryptone, sodium chloride (NaCl), yeast extract, tryptic soy agar (TSA), ampicillin sodium salt, sodium phosphate dibasic anhydrous (Na_2_HPO_4_), sodium hydroxide (NaOH), urea, dextrose monohydrate (d-glucose), magnesium sulfate (MgSO_4_), calcium chloride (CaCl_2_), cobaltous chloride hexahydrate (CoCl_2_·6H_2_O), isopropyl β-d-1-thiogalactopyranoside (IPTG), Pierce bicinchoninic acid (BCA) assay kit, Pierce snakeskin dialysis tubing 3.5 K molecular weight cutoff (MWCO), sodium dodecyl sulfate (SDS), Nunc ninety-six well plates, and BD Clay Adams glass microscopy slides were acquired from Thermo Fisher Scientific. The 20 naturally occurring amino acids, dimethyl sulfoxide (DMSO), N95 Amine (2-Amino-2-methyl-1-propanol), and thiamine hydrochloride (vitamin B) were purchased from Sigma-Aldrich. Hydrochloric acid (HCl) and Coomassie Brilliant Blue G-250 were purchased from VWR. HiTrap FF 5 mL columns for protein purification were purchased from Cytiva Life Sciences. Macrosep and Microsep Advance Centrifugal Devices 3K MWCO and 0.2 μm syringe filters were purchased from PALL. Acrylamide/bis solution (30%) 29:1, and natural polypeptide sodium dodecyl sulfate–polyacrylamide gel electrophoresis (SDS-PAGE) standard were purchased from Bio-Rad. Imidazole were purchased from Acros Organics. Formvar/carbon-coated copper grids (FCF400-Cu) and 1% uranyl acetate for transmission electron microscopy were purchased from Electron Microscopy Sciences. CELLOSIZE Hydroxyethyl Cellulose PCG-10 was obtained from the Dow Chemical company while Stearyl Alcohol, Cetearyl Alcohol, and Coconut Oil were obtained from Making cosmetics. Capric/Caprylic Triglycerides, Glyceryl Stearate (and) PEG-100 Stearate were acquired from Croda while Glycerin was purchased from Sigma-Aldrich

### Electrostatic Modeling

Q5 protein was modeled for its electrostatic potential maps as described previously for protein, Q.^20^ Rossetta (2020 release) was used to generate 500 full length poses of Q5 (AA1–54 included) using the Relax protocol and symmetry derived from COMPcc (PDB: 3V2P). The best-scoring pose was selected for Poisson–Boltzmann electrostatics calculations. PDB 2PQR (version 3.1.0) was used to titrate the Q5 pose to pH 6.0 and 8.0 and to calculate pKas and the isoelectric point. Subsequent PDB 2PQR outputs were used for Adaptive Poisson–Boltzmann Solver (APBS) software (version 3.0.0) to generate electrostatic potential maps in PyMOL (version 2.5.4).^22^

### Q5 Expression and Purification

Expression of Q5 was performed as previously described using supplemented M9 media.^13,15^ Briefly, pQE60/Q5 plasmid was transformed into chemically competent M15MA cells on TSA plates prior to inoculating 16 mL starter cultures in minimal M9 media supplemented with selected colonies, the 20 canonical amino acids, ampicillin, kanamycin, and vitamin B. Starter cultures were used to inoculate 400 mL of supplemented M9 media and was induced with IPTG once optical densities at 600 nm (OD600) was measured to be ∼0.8. Following 3 h, cells were harvested by centrifugation for 30 min at 4000*g* at 4 °C in an Avanti J-25 centrifuge (Beckman Coulter). Q5 was purified as previously described by lysing cells with a Q500 probe sonicator (QSonica, Newtown, CT), removing cell debris by centrifugation, and purifying via immobilized metal affinity chromatography in a syringe pump (Harvard Instruments, Holliston, MA) equipped with a cobalt-charged HiTRAP FF 5 mL column. Pure protein was confirmed using 12% sodium dodecyl sulfate polyacrylamide gel electrophoresis (SDS-PAGE) (Figure S3) and dialyzed in 6 consecutive buckets of 50 mM TrisHCl, 500 mM NaCl at pH 8.0 buffer at room temperature. Protein was then concentrated and buffer exchanged with 50 mM TrisHCl, 500 mM NaCl at pH 6.0 buffer for protein samples tested at pH 6.0. Protein concentration was subsequently determined by a bicinchoninic acid (BCA) assay with a standard curve based on bovine serum albumin concentrations.

### Secondary Structure Assessment

Secondary structure and deconvolution of pre- and post- gelation Q5 variants were assessed by circular dichroism (CD) spectra on a Jasco J-815 CD spectrometer equipped with a PTC-423S single position Peltier temperature control system (Jasco, Easton, MD) and attenuated total reflectance-Fourier transform infrared (ATR-FTIR) spectra on a Nicolet 6700 Fourier Transform Infrared Spectrometer equipped with a mercury cadmium telluride (MCT)-A detector as described previously.^13^ CD measurements were performed using 15 μM final concentrations of Q5 diluted in water, and ATR-FTIR measurements were performed at 2 mM in 50 mM TrisHCl, 500 mM NaCl at pH 6.0. Measurements made of the solution state Q5 hydrogel were performed immediately following concentration, whereas measurements made of the gel state Q5 were performed after confirming gelation by microrheology following incubation at 4 °C.

### Transmission Electron Microscopy

Transmission electron microscopy (TEM) images of Q5 fibers at pH 6.0 and 8.0 were taken with a FEI Talos L120C transmission electron microscope as described previously.^13,15^ Briefly, 3 μL of 50 μM protein was spotted on Formvar/carbon-coated copper grids, washed with 5 μL of deionized water, and stained with 3 μL of 1% uranyl acetate with incubation times of 1 min.

### Coformulation of Q5 with Skin Cream Chassis

The skin cream chassis was prepared as an oil in water emulsion with the ingredients listed in Table S1. Following dialysis at room temperature to remove excess imidazole, Q5 was buffer exchanged into a 5× volume of Tris-buffered saline buffer (50 mM Tris, 500 mM NaCl) at either pH 6.0 or 8.0. In the swapped buffers, pH was adjusted using N95 amine (2-amino-2-methyl-1-propanol) to mimic the relevant pH range in PSCPs. Q5 protein solution was mixed with skin chassis so the final formulation (Table S1) possessed 2 mM protein. Afterward, 100 μL of Q5 protein concentrated to 4 mM at the proper pH buffer were added to 100 μL of skin chassis in a 2 mL low-binding microcentrifuge tube. Negative control samples of SC chassis formulated with the same volume of buffer without protein were created. Samples were incubated at 4 °C for 1 week to allow Q5 to undergo a sol–gel transition by passing through its UCST.

### Titration

Q5 protein at 2 mM was buffer exchanged to pH 6.0 in a Tris-buffered saline buffer (50 mM Tris, 500 mM NaCl). The formulation was next titrated using either N95 amine (2-amino-2-methyl-1-propanol) or HCl to assess the immediate change of pH with respect to base and acid titration, respectively, using a Mettler Toledo FiveEasy F20 pH-meter equipped with a Mettler Toledo LE438 pH probe.

### Tube Inversion

Binary sol–gel behavior was assessed using tube inversion.^13,15^ Immediately after concentration to 2 mM, 150 μL of Q hydrogel variants were incubated in 2 mL microtubes at 4 °C for 2 weeks. Gelation was visually observed by inverting the microtube and inspecting the sample for flow from the top of the tube where weak or strong gel-like behavior was considered a gel.

### Rheological Assessment

Gelation rates of Q5 at pH 6.0 and pH 8.0 were assessed using passive microrheology at 4 °C, described previously.^19^ Briefly, 2 mM samples of Q5 were aliquoted with 1% v/v 1 μm diameter FluoSpheres inside a glass capillary tube (VitroCom) and imaged periodically using an inverted fluorescent microscope (ZEISS Microscopy) at 40× magnification with 2 × 2 binning while being incubated at 4 °C on a rotisserie at 8 rpm between measurements. Rheology of Q5 and Q5 coformulations were measured using a stress-controlled rheometer (Discovery Hybrid Rheometer 2, TA Instruments) equipped with a 8 mm parallel plate geometry. Q5 coformulations were measured after mixing with skin cream chassis, and Q5 alone was measured at pH 6.0 and pH 8.0 conditions after incubation at 4 °C for 1 week. Rheology measurements were made using a frequency sweep from 0.1 to 10 Hz, where the linear viscoelastic region (LVR) was reported between 1 and 10 Hz, at 4 °C and an oscillation strain of 5% and a gap size of 200 μm.

### Statistical Analysis

Our data of three replicates was represented as mean ± standard deviation. Prism 10 (GraphPad software, MA, USA) was employed for statistical analysis using student’s *t*-test and 2-way ANOVA analysis.

## Results and Discussion

### pH-Dependent Electrostatics and Nanoassembly

**Figure 1:**
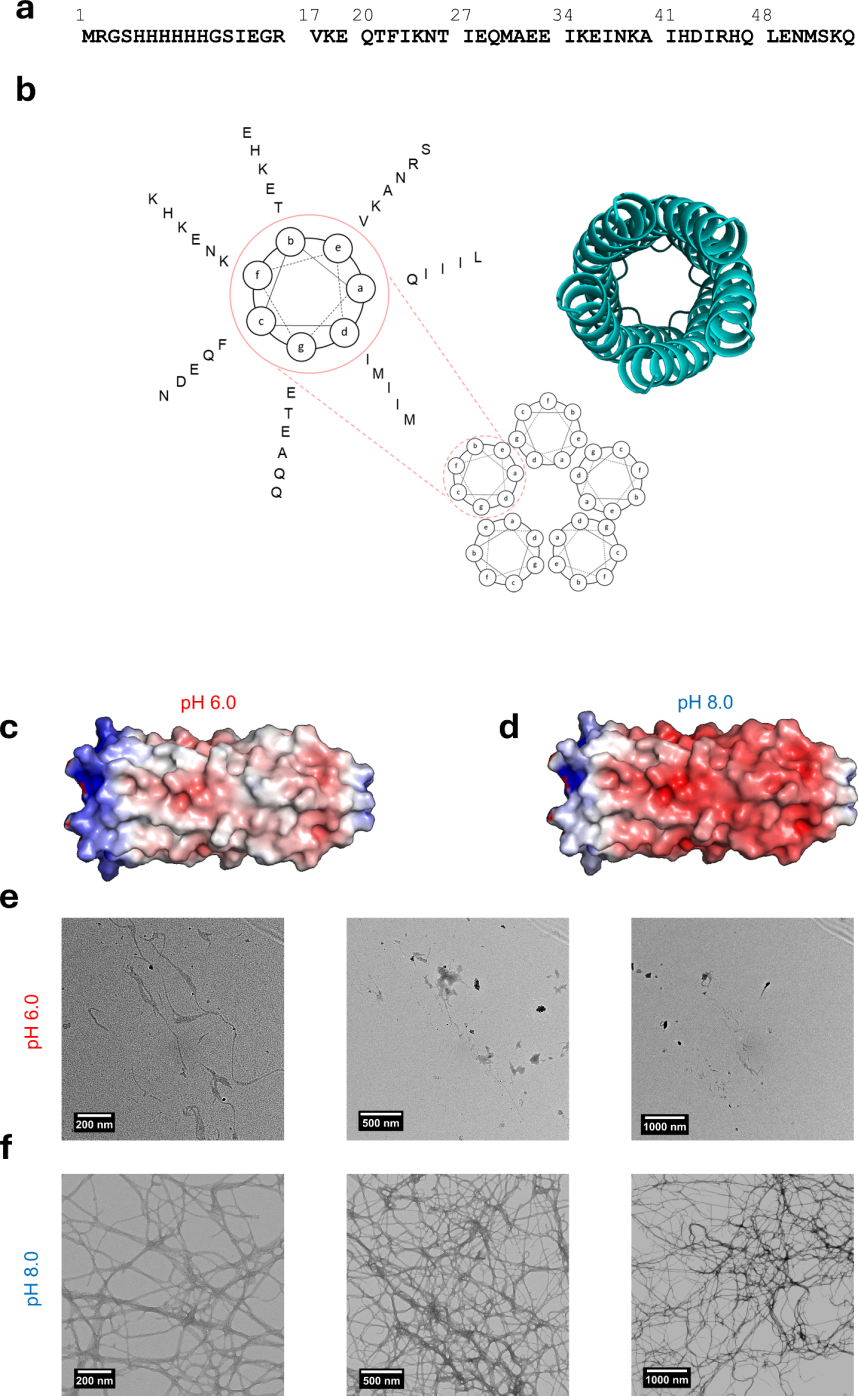
(a) Full-length protein sequence for Q5. (b) Helical wheel diagram of the pentameric coiled coil with a cartoon ribbon diagram of Q5 for reference. One helical wheel of Q5 is highlighted with corresponding helical wheel positions matching location of residues in order from closer to the helical wheel to the outside beginning with partial heptad (VKE) starting at the *e* helical wheel position. Electrostatic potential energy maps of Q5 calculated at (c) pH 6.0 and (d) pH 8.0. Transmission electron micrographs of Q5 at (e) pH 6.0 and (f) pH 8.0 at 45 kX, 22 kX, and 11 kX resolutions from left to right. Helical wheel diagram and ribbon cartoon of Q5 in panel b is adapted from Figure 1 panel a in Britton et al., *ACS Applied Bio Materials*, 2024.^24^

Q5 was previously designed using a probabilistic Rosetta-score based Monte Carlo search.^13^ Briefly, Q was used as an input sequence and was allowed to be mutated for up to 500 attempts before selecting a pose with the best Rosetta-score as a proxy for improved protein stability. This was repeated 60 times before mapping each best-scoring sequence to the probability that a mutation existed at a given sequence position, resulting in Q5 (Figure 1a). Residues attributed to electrostatic interaction and fiber assembly are those located in the *b*, *c*, and *f* helical wheel positions and residues attributed to hydrophobic coiled-coil formation are located in the *a* and *d* helical wheel positions (Figure 1b) where coiled-coil supersecondary structure follows a heptad pattern denoted by positions *a* through *g*.^23^ The Q5 protein was further characterized to possess the increased material strength, α-helical structure, and gelation time that was well-correlated to thinner fiber assemblies resulting in denser physical cross-linking.^13^ We have also previously established the parent Q sequence to possess pH dependence where pH closer to the sequence pI possessed increased fiber formation, material strength, and gelation times.^19,20^ Q5 possessed a pI of 8.0 (Figure S1), substantially lower than Q which possessed a pI of 10.3.^20^ Dynamics of pH-responsiveness were further assessed by titrating with HCl or N95 amine, which resulted in an immediate pH response and an expected neutral equivalence point (Figure S2). Thus, we expected Q5 to be a better candidate to resist a loss of gelation when incubated at the lower pHs required for skin ointments.

To explore the impact of pH on the ability for Q5 to form complementary charges necessary for end-to-end stacking, confirmed recently by molecular dynamics (MD) modeling and SAXS,^13^ electrostatic potential energy maps were generated from Rosetta models and PDQ2PQR-APBS software at pH 6.0 and pH 8.0 (Figure 1c) and compared to previous electrostatic potential energy maps of Q.^20^ Q5 revealed substantially increased negative charge on its surface in comparison to Q at both pH 6.0 (Figure 1c) and pH 8.0 (Figure 1d), marked by increased intensity of red on the C-terminus. At pH 6.0 and pH 8.0, Q5 possessed a smaller area of positive surface charge on the N-terminus in comparison to Q. In consideration of the positively charged N-terminal histag, this is critical to the lower pI of the sequence and ability for Q5 to maintain a lower electrostatic potential energy difference of surface residues necessary for thin fiber formation.^13^ To this extent, at pH 6.0 (Figure 1c), Q5 maintains a substantially less positively charged patch at the N-terminus compared to Q, suggesting an increased likelihood to form fibers at lower pH.^20^

To study the relative gelation and nanoscale assembly of Q5 at different pH, Q5 was successfully expressed and purified as done previously (Figure S3).^13^ After incubation of Q5 at 4 °C, Q5 was observed to form a viscous gel-like material at pH 6.0 whereas at pH 8.0, Q5 was observed to form a strong gel material, consistent with previous studies.^13^ In all studies, we use 2 mM (1.3 w/w %) Q5 in Tris-buffer saline (50 mM Tris, 500 mM NaCl) and allow for hydrogel formation at 4 °C. We have previously established the concentration and temperature dependence through generating independent samples of Q5 at different concentrations and temperatures. Using a bivariate linear regression analysis we have determined Q5 to possess a concentration dependence of 0.237 η·mM^–1^ and a temperature dependence of 0.032 η·°C^1–^ where the extent of gelation is represented by normalized value, η, on a scale of 0 (solution-like behavior) to 1 (gel-like behavior).^13^

To compare the gel properties at different pH, transmission electron microscopy (TEM) was employed to assess the ability for Q5 to form nanoscale fibers at pH 6.0 (Figure 1e) and pH 8.0 (Figure 1f, Figure S4) after incubation at 4 °C. At pH 8.0, dense networks of physically cross-linked fibers were formed consisting of thin nanofibers. In some populations, shearing effects due to sample dilution demonstrated that a population of small groupings of cross-linked fibers form (Figure S4). We consider this an important behavior that is likely to occur when mixing with SC chasses. In comparison, at pH 6.0, Q5 exhibited the formation of some nanofibers and had relatively few physical cross-links with each other. This difference suggested that supramolecular assembly was hindered by the low pH. However, the formation of some nanofibers was consistent with the visual appearance of a viscous material and was a substantial difference compared to the behavior of the parent, Q, at pH 6.0. Previously, Q was shown to be unable to form nanofibers and results in a watery solution at pH 6.^19,20^ Furthermore, TEM images of Q revealed the formation of dispersed aggregates alone,^20^ whereas Q5 demonstrated a population of both dispersed aggregates and thin nanofibers.

### pH-Dependent Protein Rheology

**Figure 2:**
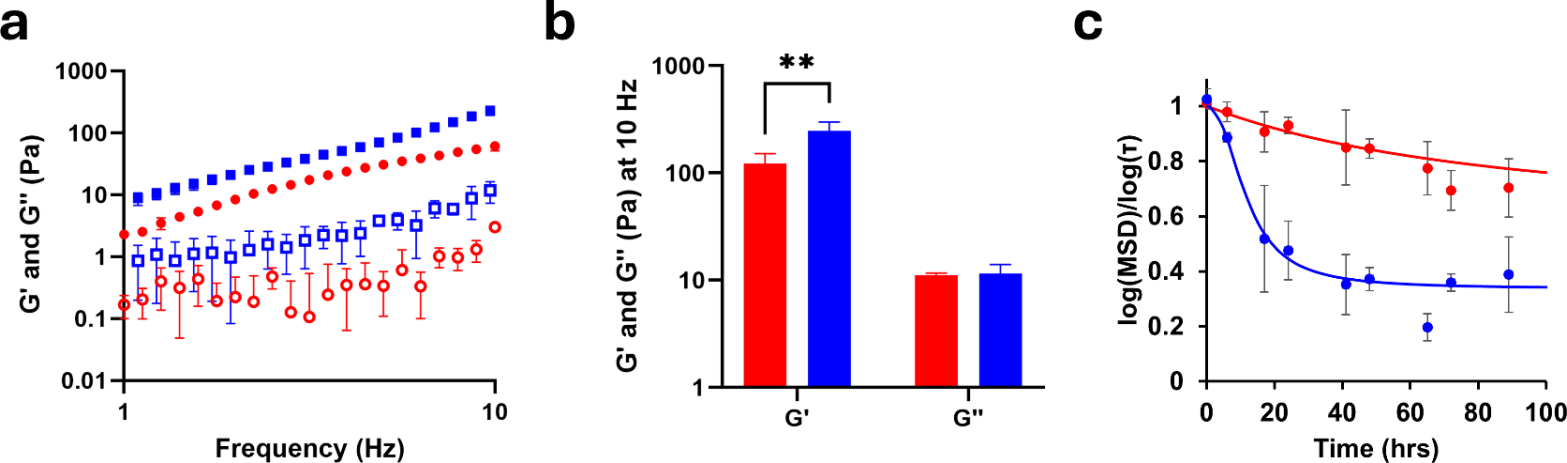
(a) Rheology was measured as a frequency sweep of Q5 at 2 mM at pH 6.0 (red) and pH 8.0 (blue) for average storage modulus (*G*′, filled markers) and average loss modulus (*G*″, empty markers). (b) Average *G*′ and *G*″ of Q5 at 2 mM using 10 Hz frequency at pH 6.0 (red) and pH 8.0 (blue). (c) Sigmoidal fit for Q5 at 2 mM at pH 6.0 (red) and 8.0 (blue) of average microrheologically determined logarithmic slopes of MSD-τ. Error bars represent the standard deviations of three independent trials. Data for rheology measurements of Q5 at pH 8.0 are adapted from Britton et al., *Biomacromolecules*, 2024.^13^

A parallel plate rheometer was employed to confirm the rheological behavior of Q5 as a gel at pH 6.0 and 8.0 (Figure 2a). Due to the instrument low-torque limit and inertia effect,^25^ we were unable to reach low frequencies (Figure S5). In the interest of exploring suspensions of skin emulsions with protein rheological modifiers, higher frequencies were also not explored. Thus, we report rheology of our materials between 1 and 10 Hz. Additionally, the crossover modulus was observed between 0.1 to 1 Hz, where the linear viscoelastic region (LVR) representative of Q5 molecular interactions is clear between 1 and 10 Hz (Figure S5). Q5 exhibited elastic behavior at pH 6.0 and pH 8.0 in the LVR, consistent with soft hydrogel formation, possessing storage modulus (*G*′) > loss modulus (*G*″) at all frequencies studied at 4 °C. In contrast, parent Q did not result in gel formation studied in standard parallel plate rheometry.^20^ Q5 demonstrated a loss of material strength at pH 6.0 compared to pH 8.0 indicated by a slight loss of *G*′ from 1 to 10 Hz. As a standard comparison for our Q hydrogel system,^13,15,20,26^ we evaluated the *G*′ and *G*″ at 10 Hz, which revealed Q5 to possess a significantly increased *G*′ at pH 8.0 of 246 ± 53 Pa compared to at pH 6.0 of 122 ± 30 Pa (Figure 2b, Table 1). Q5 did not show a significant difference in *G*″ at 10 Hz with values of 11 ± 1 and 11 ± 2 Pa at pH 6.0 and 8.0, respectively (Figure 2b, Table 1). Overall, Q5 showed resilience to pH-dependent transition to a solution at low pH, demonstrating a slight loss compared to Q5 at pH 8.0, but a greater *G*′ in comparison to the Q parent at pH 6.0 (no gel) and even at pH 8.0 (50.4 Pa). The ability for Q5 to form a weak gel at pH 6.0 suggested that its fiber formation (Figure 1e) was sufficient to form a physically cross-linked hydrogel.

**Table 1:** Average and Standard Deviation of Storage Modulus (*G*′) and Loss Modulus (*G*″) Measured by a Parallel Plate Rheometer for SC, SC+Q5, SC+HEC, and SC+Q5+HEC; Data for Rheology Measurements of Q5 at pH 8.0 is Adapted from Britton et al., *Biomacromolecules*, 2024^13^

			Skin Ointment Formulation			
		Q5	SC	SC+Q5	SC+HEC	SC+Q5+HEC
pH 6.0	*G*′ at 10 Hz	122 ± 30 Pa	126 ± 33 Pa	275 ± 87 Pa	194 ± 44 Pa	218 ± 33 Pa
	*G*″ at 10 Hz	11 ± 1	13 ± 1 Pa	25 ± 20 Pa	24 ± 9 Pa	13 ± 6 Pa
pH 8.0	*G*′ at 10 Hz	246 ± 53	237 ± 35 Pa	175 ± 34 Pa	204 ± 65 Pa	122 ± 6 Pa
	*G*″ at 10 Hz	12 ± 2	24 ± 1 Pa	14 ± 2 Pa	17 ± 5 Pa	17 ± 2 Pa

To evaluate the kinetics of the solution-to-gel (sol–gel) transition, passive microrheology was employed using 2 mM samples of Q5 mixed with fluorescent tracer beads (FluoSpheres) at pH 6.0 and pH 8.0. Samples were incubated at 4 °C and measured periodically for ∼4 days. MSDs were subsequently calculated by multiple particle tracking (MPT) and gelation times were determined by calculating logarithmic slopes of the mean square displacement (MSD) with respect to lag time (τ) (Figure 2c). We previously determined the critical gelation times (t_c_) of Q5, as analyzed by time-cure superposition, to be 11.5 ± 1.5 h at pH 8.0. At pH 6.0, Q5 did not exhibit a complete sol–gel transition and instead demonstrated partial transition into a hydrogel with some decrease in the logarithmic slopes of the MSD-τ. Logarithmic slopes of the MSD-τ < 1 are considered subdiffusive behavior and a transition from Brownian motion of particles represented by values of ∼1.^27^ Using sigmoidal analysis to assess master solution states of the hydrogels, Q5 revealed significant differences in the plateaus of the logarithmic slopes of the MSD-τ. Using a sigmoidal fit, Q5 at pH 6.0 was measured to plateau at a slope of 0.66 ± 0.06, indicating partial transition into a hydrogel and consistent with its viscoelastic nature at pH 6. In comparison, Q5 at pH 8.0 was measured to plateau at 0.28 ± 0.14, indicating mostly gel-like behavior and consistent with our observation of its complete transition into a hydrogel.^13^

### pH-Dependent Secondary Structure of Q5

**Figure 3:**
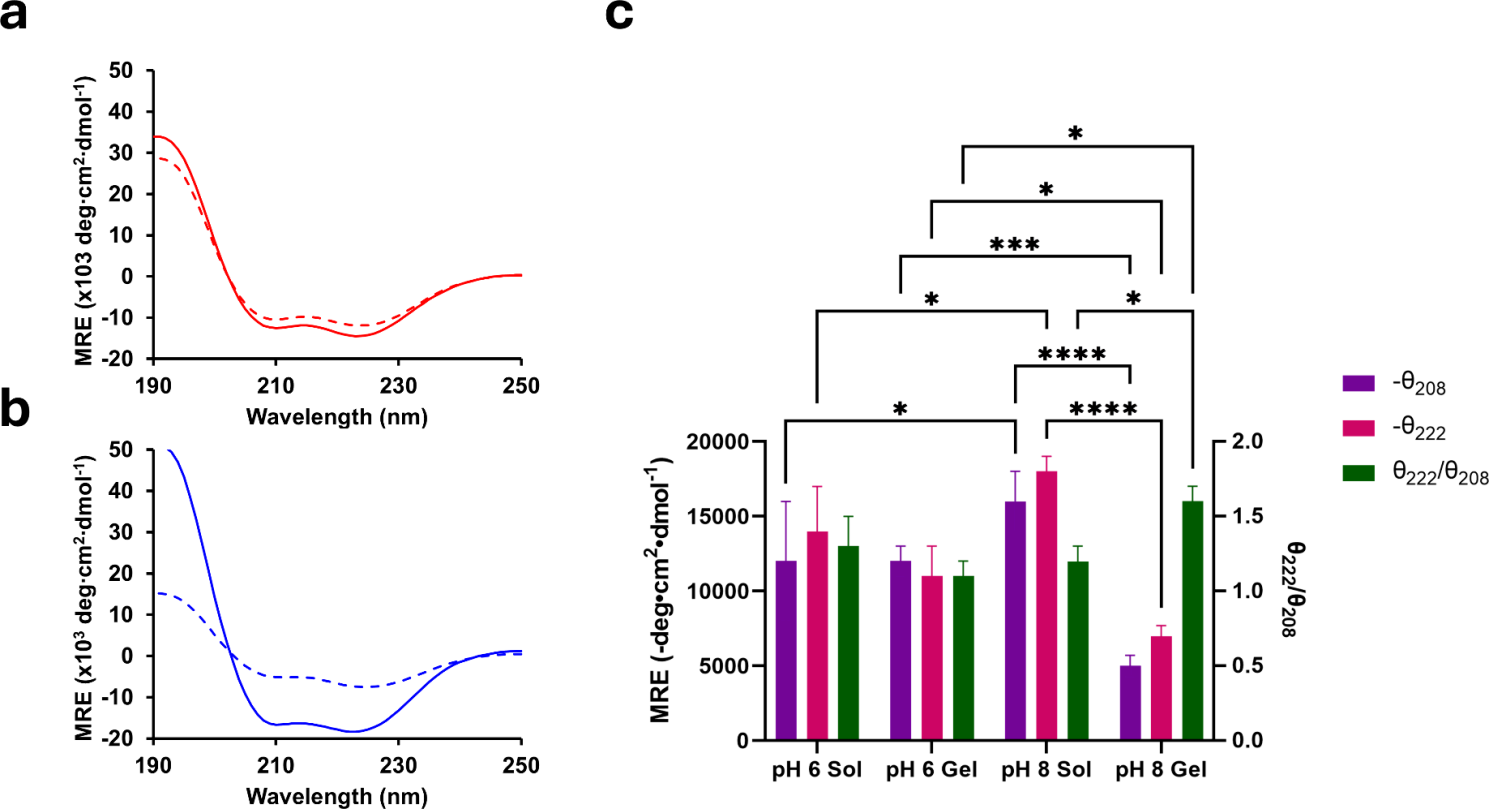
Average CD wavelength scans at 4 °C of Q5 in the solution state (solid line) and gel state (after incubation at 4 °C, dashed line) at (a) pH 6.0 and (b) pH 8.0. (c) Average MRE for helical double minima of Q5 at 208 nm (purple) and 222 nm (magenta) and average ratio of MRE at 208 and 222 nm (green) from pH 6.0 and pH 8.0 CD measurements. Error bars represent the standard deviation of three independent trials. Statistical tests are shown for measurements compared at same solution/gel states and/or same wavelength/ratios. * represents *p*-value <0.05, *** represents *p*-value <0.001, **** represents *p*-value <0.0001. Data for circular dichroism measurements of Q5 at pH 8.0 in panel b and c is adapted from Britton et al., *Biomacromolecules*, 2024.^13^

To assess the secondary structure of Q5, CD measurements were performed at pH 6.0 and 8.0 before and after incubation at 4 °C (Figure 3). Immediately after concentration to 2 mM, Q5 was subjected to CD wavelength scans diluted in water to 15 μM to assess secondary structure in the solution state prior to incubation at 4 °C (Figure 3a). Q5 exhibited strong α-helical secondary structure at both pH 6.0 and pH 8.0, possessing a double minima at 208 and 222 nm. At pH 6.0, Q5 showed a double minima of −14,000 ± 3,000 deg·cm^2^·dmol^–1^ and −12,000 ± 4,000 deg·cm^2^·dmol^–1^ at 208 and 222 nm, respectively (Figure 3a). This represented a statistically significant loss compared to Q5 at pH 8.0 which was measured to possess a double minima of −16,000 ± 2,000 deg·cm^2^·dmol^–1^ and −18,000 ± 1,000 deg·cm^2^·dmol^–1^ at 208 and 222 nm, respectively^13^ (Figure 3b, c, Table S2).

Following, Q5 was incubated at 4 °C to allow for transition into the gel state that was subsequently measured by CD wavelength scans diluted in water to 15 μM (Figure 3b). Previously, Q5 was measured to undergo a significant signal dampening after gelation at pH 8.0, possessing a double minima of −5,000 ± 1,000 deg·cm^2^·dmol^–1^ at 208 nm and −7,000 ± 1,000 deg·cm^2^·dmol^–1^ and 222 nm, marking a 69% and 61% signal loss, respectively^13^ (Figure 3c, Table S2). In contrast, when incubated at 4 °C at pH 6.0, Q5 showed a negligible loss of signal of −12,000 ± 1,000 deg·cm^2^·dmol^–1^ at 208 nm and −11,000 ± 2,000 at 222 nm, representing a 0% and 21% signal loss, respectively (Figure 3c, Table S2). Previously, we have noted that signal dampening in CD was associated with a transition into a hydrogel.^13^ The smaller loss of signal revealed by Q5 at a lower pH was consistent with its weaker material strength.

The stronger signal of the gel state at lower pH was also consistent with previous pH-dependence studies of the parent protein, Q,^20^ which demonstrates a greater magnitude of the double minima with signals of −15,000 deg·cm^2^·dmol^–1^ at 208 nm and −9,000 deg·cm^2^·dmol^–1^ at 222 nm, at pH 6.0,^20^ compared to a double minima of −5,000 deg·cm^2^·dmol^–1^ at 208 nm and −4,000 deg·cm^2^·dmol^–1^ and 222 nm, for Q5,^17^ representing a 300% increase at 208 nm and 225% increase at 222 nm (Figure 3c, Table S2). Q5 also exhibited a statistically significant increase in the signal of the gel state at pH 6.0 with increases of 240% and 157%, respectively. The smaller increase in signal of Q5 compared to Q also supported previous signal dampening results associated with transition into the gel state.^13^ Q5 was still capable of some transition into the gel state at pH 6.0 (Figure 2a) whereas Q did not exhibit gelation at pH 6.0.^20^

Q5 also demonstrated a statistically significant increase in its 222/208 ratio from the solution state to the gel state at pH 8.0, from 1.2 ± 0.1 to 1.6 ± 0.1 (Figure 3c, Table S2), respectively, suggesting an increase in the relative amount of helices packed closely together rather than in isolation.^28−30^ In comparison, Q5 revealed a negligible loss in its 222/208 ratio from the solution state to the gel state at pH 6.0, from 1.3 ± 0.2 to 1.1 ± 0.1, respectively (Figure 3c, Table S2). A statistically significant difference was also present in comparison of Q5 in the gel state at pH 6.0 to 8.0, where there was a slight increase at pH 8.0, suggesting an increase in the packing of helices together.

**Figure 4:**
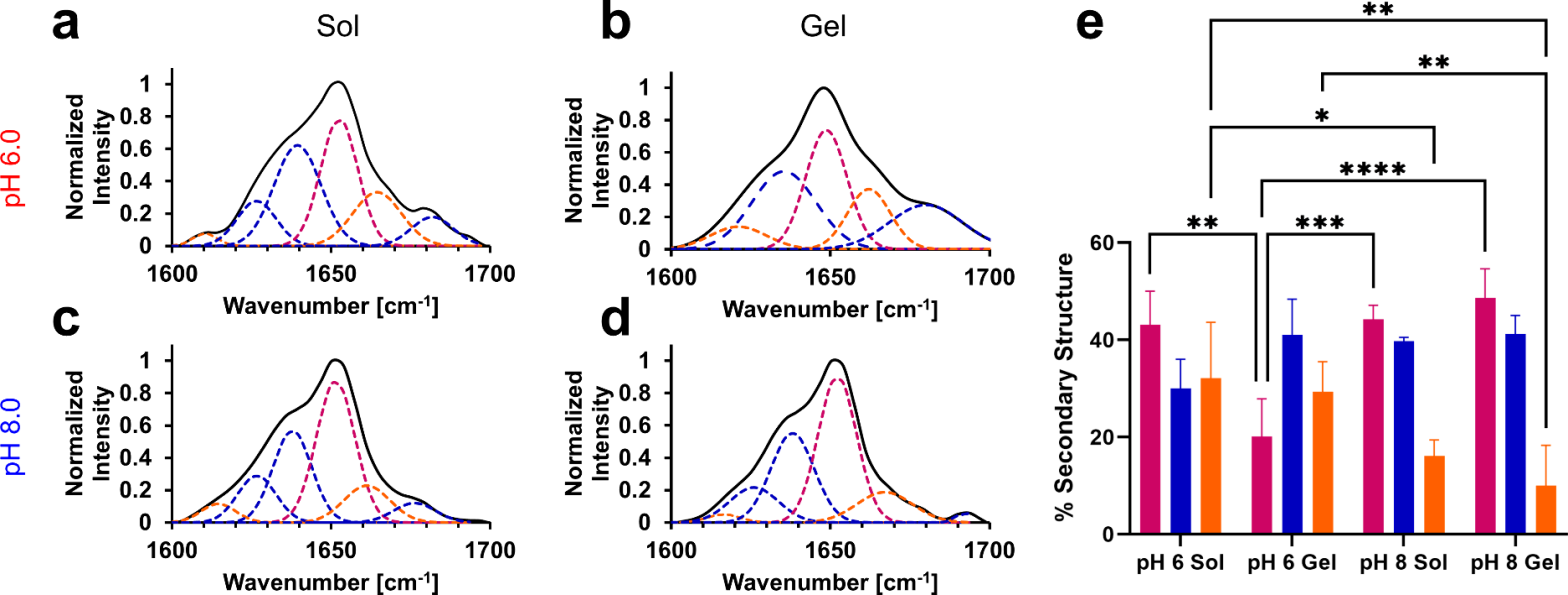
Representative ATR-FTIR spectra of the amide I bond region for Q5 at pH 6.0 in the (a) solution (sol) state and the (b) gel state and at pH 8.0 in the (c) solution state and the (d) gel state. (e) Average deconvoluted secondary structure for Q5 at pH 6.0 and pH 8.0 in the solution state and gel state. Deconvolution of secondary structure from ATR-FTIR spectra are color-coded as magenta: α-helix, blue: β-sheet, and orange: random coil. Error bars represent the standard deviation of three independent trials. * represents *p*-value <0.05, ** represents *p*-value <0.01, *** represents *p*-value <0.001, **** represents *p*-value <0.0001. Data for ATR-FTIR spectra and associated deconvolution of Q5 at pH 8.0 in panel c, d, and e is adapted from Britton et al., *Biomacromolecules*, 2024.^13^

To evaluate the secondary structure of Q5 at various pH values in its representative buffer and concentration, attenuated total reflectance-Fourier transform (ATR-FTIR) spectroscopy was employed to allow for independent secondary structure deconvolution (Figure 4). At pH 6.0, Q5 exhibited a general broadening of the amide I bond region from the solution (sol) state (Figure 4a) to gel state (Figure 4b), indicating loss of α-helical structure. Shifting of peaks toward lower wavenumbers at pH 6.0 was also consistent with ATR-FTIR measurements in Q previously at lower pH.^20^ In comparison, strong helical peaks were confirmed for Q5 at pH 8.0 in both the sol state (Figure 4c) and gel state (Figure 4d). These differences represented a statistically significant decrease in relative helicity of Q5 in the gel state at pH 6.0 from 43.1 ± 6.9% to 20.2 ± 7.7% (Figure 4e, Table S2). At pH 8.0, no significant differences were observed between the secondary structures measured in the sol state and the gel state of Q5 (Figure 4e, Table S2).

Q5 also exhibited large differences in overall secondary structure when comparing the solution or gel states at a given pH. Q5 possessed increased structured content in the sol state at pH 8.0 in comparison to pH 6.0, represented by a decrease in random coil content from 32.1 ± 11.5% to 16.1 ± 3.3%, respectively (Figure 4e, Table S2). This difference increased further upon transition into a hydrogel where, at pH 8.0, Q5 possessed 10.1 ± 8.3% random coil content, a substantial decrease compared to Q5 at pH 6.0, which possessed 29.3 ± 6.2% random coil content (Figure 4c, Table S2). We associate the increased α-helical secondary structure content and decreased random coil content of Q5 at pH 8.0 compared to Q5 at pH 6.0 was associated with its increased propensity for ordered supramolecular assembly (Figure 1e–f) and gel formation (Figure 2).

### Q5 as a pH-Dependent Rheological Modifier in Skin Ointment

**Figure 5:**
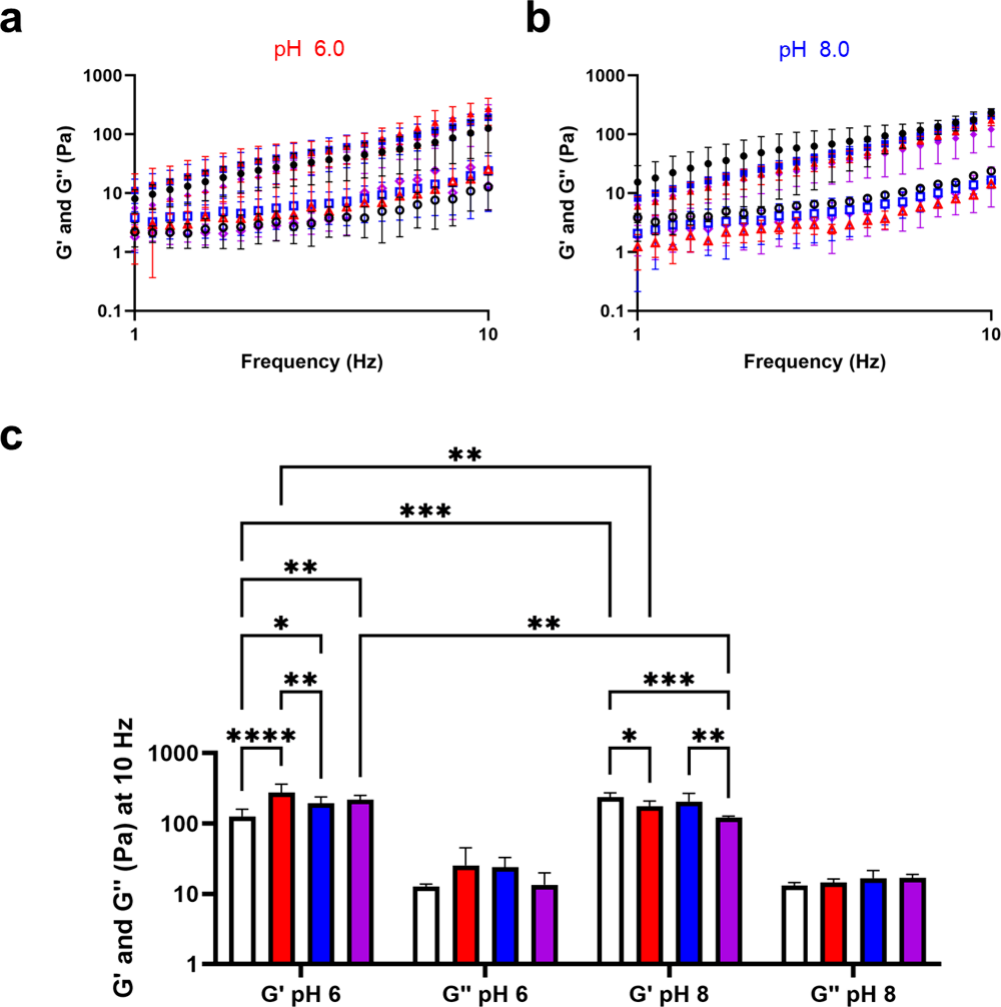
Rheology measured as a frequency sweep of SC (black), SC+Q5 (red), SC+HEC (blue), and SC+Q5+HEC (purple) at (a) pH 6.0 and (b) pH 8.0 for average storage modulus (*G*′, filled markers) and average loss modulus (*G*″, empty markers). (c) Average *G*′ and *G*″ at 10 Hz for SC (white), SC+Q5 (red), SC+HEC (blue), and SC+Q5+HEC (purple) at pH 6.0 and pH 8.0. Error bars represent the standard deviation of three independent trials. Statistical tests are shown for coformulations at the same pH or between the same coformulation at pH 6.0 vs pH 8.0. * represents *p*-value <0.05, ** represents *p*-value <0.01, *** represents *p*-value <0.001, **** represents *p*-value <0.0001.

To assess the ability to act as a rheological modifier in skin ointment, Q5 was carefully mixed in a standard skin cream chassis (SC) (Table S1) at pH 6.0 and pH 8.0 to a final concentration of 1.3% w/w, at 4 °C and compared to SC alone and with a standard rheological modifier, hydroxyethyl cellulose (HEC) at 0.2% w/w. SC, Q5 mixed with SC (SC+Q5), SC mixed with 0.2% w/w HEC (SC+HEC), and Q5 mixed in SC with HEC (SC+Q5+HEC) were assessed using a frequency sweep from 1 to 10 Hz at pH 6.0 (Figure 5a, Figure S5b) and at pH 8.0 (Figure 5b, Figure S5c), as done for Q5 alone at pH 6.0 and at pH 8.0 (Figure 2a). All formulations demonstrated *G*′ > *G*″ at both pH 6.0 and pH 8.0, indicative of gel-like behavior (Figure 5a–b, Figure S5b–c) and the SC exhibited almost identical rheological behavior in comparison to Q5 alone (Figure 2b, Figure 5c, Table 1). SC+Q5 demonstrated the strongest material strength at pH 6.0 with a *G*′ of 275 ± 87 Pa in comparison to SC, SC+HEC, and SC+Q5+HEC with values of 127 ± 33 Pa, 194 ± 44, and 218 ± 34 Pa, respectively (Figure 5c, Table 1). In contrast, all rheological modifiers seem to slightly weaken the ointment formulations where SC possessed a *G*′ at pH 8.0 of 237 ± 35 Pa compared to 175 ± 34 Pa, 204 ± 65, and 122 ± 6 Pa for SC+Q5, SC+HEC, and SC+Q5+HEC, respectively (Figure 5c, Table 1).

Interestingly, the various formulations demonstrated diverse pH-dependent rheological behavior (Figure 5c, Table 1). SC with the addition of Q5 or Q5+HEC as rheological modifiers revealed significantly increased *G*′ at pH 6.0 compared to pH 8.0, whereas SC alone revealed a significant decrease in *G*′ at pH 6.0 compared to pH 8.0 and SC+HEC demonstrated a negligible decrease at pH 6.0 compared to pH 8.0 (Figure 5c, Table 1) which is expected from nonionic polysaccharide based rheology modifier. This strongly suggested that addition of Q5 caused an increase in material strength at lower pH, a substantial difference from SC alone, which appears to possess the opposite behavior. This was also a substantial difference from HEC that caused resistance to pH-dependent rheological modification of the SC. Interestingly, the increased *G*′ at pH 6.0 compared to pH 8.0 of SC+Q5+HEC indicated that the impact of Q5 was dominant compared to HEC on the rheological behavior, where the *G*′ was expected to remain similar if HEC possessed a dominant rheological modifier impact. Furthermore, the addition of Q5 provided a similar 57% and 79% increase in *G*′ compared to that of the SC alone without and with HEC, respectively. The HEC provided (i) an independent mechanism for rheological modification that was less dominant than Q5 or (ii) a rheological modification that was hindered by Q5.

Since more dispersed fiber formation is observed at pH 6.0 than at pH 8.0, it is likely that Q5 produces larger *G*′ due to sufficiently high volume fractions of fiber particles that increase flow resistance.^31^ In comparison, smaller entangled fibers of the Q5 hydrogel at pH 8.0 are likely to be sheared into large, grouped networks due to mixing with SC chassis, consistent with TEM measurements that show a boundary between the hydrogel matrix and water (Figure S4). These larger matrix groups suggest that fibers undergo agglomeration, but are likely to maintain entanglement at pH 8.0 and offer a smaller volume fraction in the chassis, providing less flow resistance. This is consistent with the end-to-end assembly behavior of the Q hydrogel system in which pH closer to the pI allows for thinner fibers from balanced electrostatic protein–protein interactions.^20^ Coiled-coil hydrogels, such as Q5, offer a unique biocompatible, rheological modifier for skin ointments that can assist resistance to losses of *G*′ caused by the acidic skin environment.

Overall, all rheological modifiers provide sufficient *G*′ within the range of pH conditions relevant to healthy human skin for ointment applications^11,12,32^ The use of Q5 as a rheological modifier, however, benefits from possessing improved material strength at pH 6.0 (more relevant to mildly acidic conditions of the corneum) where skin ointments tend to exist within the range of ∼10^2^–10^3^ Pa at 10 Hz.^33,34^ In comparison, *G*′ values of formulations measured here existed in the range of ∼100–300 Pa at 10 Hz, on the lower end of the desired range where increases in the material strength of SC+Q5 is beneficial. Furthermore, the application of PSCPs must be considered in the selection of a rheological modifier. PSCPs require viscous consistency for desirable sensory evaluation^35^ and must have optimal viscosity to maintain solubility and stability of the active agent, while also feeling comfortable for the user.^36^ Macromolecules with the ability to elevate the material strength of PSCPs are critical to enhance structural integrity of the chassis and prolong its shelf life, thereby obtaining optimal performance.^4^ Protein-based macromolecules, such as Q5, offer the added benefit of sequence modularity. In particular, the Q hydrogel system has been established to possess strong sequence-to-rheological function relationships determined through in silico modeling and design.^13,14^ Thus, protein systems, such as Q, may be designed for specific rheological modifier profiles in PSCPs.

## Conclusions

Protein-based rheological modifiers for PSCPs offer a uniquely modular and biocompatible approach based on their amino acid composition. Coiled-coil hydrogels, such as Q5, possess the added advantage of pH-responsiveness, where pH affects its supramolecular assembly and morphology. Due to the mildly acidic environment of the skin, rheological modifiers that help maintain or enhance the chassis material strength at low pH are particularly beneficial. Due to the low pI of Q5, and the ability for coiled-coils to have increased supramolecular assembly close to the pI, we have assessed Q5 for its gelation and structural properties at pH 6.0 and pH 8.0. Q5 exhibits the ability to undergo a sol–gel transition at pH 6.0 where the parent Q, with a pI of 10.3, did not. Addition of Q5 to a standard SC with and without a standard rheological modifier, HEC, demonstrates unique pH-responsiveness. Where SC+HEC does not result in a pH-responsive change to *G*′ which is expected, SC with Q5 revealed an increased *G*′ at pH 6.0 compared to all coformulations at pH 6.0 or pH 8.0, suggesting Q5 acts as a beneficial rheological modifier for increased viscous behavior at low pH. The results of this study will help inform the design of future protein-based self-assembled rheological modifiers to have optimal pI and electrostatic potential.

## Supporting Information Available

Figures for 12% SDS-PAGE gel showing pure Q5 protein after biosynthesis, titration of Q5 using HCL and N95 amine, charge calculation of folded and unfolded Q5 protein as a coiled-coil, TEM images of Q5 showing separated physically cross-linked groups. Tables for CD double minima values and ATR-FTIR secondar structure following deconvolution, skin chassis formulation composition. (PDF)

## References

[ref1] Khalid M.; Abdollahi M. Environmental Distribution of Personal Care Products and Their Effects on Human Health. Iran J. Pharm. Res. 2021, 20 (1), 216–253. 10.22037/ijpr.2021.114891.15088. PMC8170769 34400954

[ref2] Barel A. O.; Paye M.; Maibach H. I. Handbook of Cosmetic Science and Technology, 4th ed.; Taylor & Francis, 2014.

[ref3] Tadros T. F. Formulations: In Cosmetic and Personal Care; De Gruyter, 2016.

[ref4] Zheng Y. J.; Loh X. J. Natural rheological modifiers for personal care. Polym. Adv. Technol. 2016, 27 (12), 1664–1679. 10.1002/pat.3822.

[ref5] Lupo M. P.; Cole A. L. Cosmeceutical peptides. Dermatologic Therapy 2007, 20 (5), 343–349. 10.1111/j.1529-8019.2007.00148.x. 18045359

[ref6] Faria-Silva C.; Ascenso A.; Costa A. M.; Marto J.; Carvalheiro M.; Ribeiro H. M.; Simões S. Feeding the skin: A new trend in food and cosmetics convergence. Trends in Food Science & Technology 2020, 95, 21–32. 10.1016/j.tifs.2019.11.015.

[ref7] Sarma A.; Chakraborty T.; Das M. K. Chapter 20 - Nanocosmeceuticals: Current trends, market analysis, and future trends. In Nanocosmeceuticals, Das M. K., Ed.; Academic Press, 2022; pp 525–558.

[ref8] Dexter A. F.; Malcolm A. S.; Middelberg A. P. J. Reversible active switching of the mechanical properties of a peptide film at a fluid–fluid interface. Nat. Mater. 2006, 5 (6), 502–506. 10.1038/nmat1653. 16715085

[ref9] Yu H.; Qin L.; Zhou J. Effect of Oil Polarity on the Protein Adsorption at Oil–Water Interfaces. Langmuir 2023, 39 (30), 10701–10710. 10.1021/acs.langmuir.3c01541. 37470337

[ref10] Schmid-Wendtner M. H.; Korting H. C. The pH of the Skin Surface and Its Impact on the Barrier Function. Skin Pharmacology and Physiology 2006, 19 (6), 296–302. 10.1159/000094670. 16864974

[ref11] Fluhr J. W.; Menzel P.; Schwarzer R.; Kaestle B.; Arens-Corell M.; Praefke L.; Tsankov N. K.; Nikolaeva D. G.; Misery L.; Darlenski R. Acidic Skin Care Promotes Cutaneous Microbiome Recovery and Skin Physiology in an Acute Stratum Corneum Stress Model. Skin Pharmacology and Physiology 2022, 35 (5), 266–277. 10.1159/000526228. 35908536

[ref12] Hawkins S.; Dasgupta B. R.; Ananthapadmanabhan K. P. Role of pH in skin cleansing. International Journal of Cosmetic Science 2021, 43 (4), 474–483. 10.1111/ics.12721. 34137035

[ref13] Britton D.; Christians L. F.; Liu C.; Legocki J.; Xiao Y.; Meleties M.; Yang L.; Cammer M.; Jia S.; Zhang Z.; et al. Computational Prediction of Coiled–Coil Protein Gelation Dynamics and Structure. Biomacromolecules 2024, 25 (1), 258–271. 10.1021/acs.biomac.3c00968. 38110299 PMC10777397

[ref14] Britton D.; Legocki J.; Paul D.; Katsara O.; Aristizabal O.; Pandya N.; Mishkit O.; Xiao Y.; Aristizabal M.; Rahman N. Coiled-Coil Protein Hydrogels Engineered with Minimized Fiber Diameters for Sustained Release of Doxorubicin in Triple-Negative Breast Cancer. ACS Biomaterials Science & Engineering 2024, 10, 3425 10.1021/acsbiomaterials.4c00349. 38622760 PMC11094684

[ref15] Britton D.; Meleties M.; Liu C.; Jia S.; Mahmoudinobar F.; Renfrew P. D.; Bonneau R.; Montclare J. K. Tuning a coiled-coil hydrogel via computational design of supramolecular fiber assembly. Molecular Systems Design & Engineering 2023, 8 (2), 217–226. 10.1039/D2ME00153E.

[ref16] Britton D.; Monkovic J.; Jia S.; Liu C.; Mahmoudinobar F.; Meleties M.; Renfrew P. D.; Bonneau R.; Montclare J. K. Supramolecular Assembly and Small-Molecule Binding by Protein-Engineered Coiled-Coil Fibers. Biomacromolecules 2022, 23 (11), 4851–4859. 10.1021/acs.biomac.2c01031. 36227640

[ref17] Hill L. K.; Meleties M.; Katyal P.; Xie X.; Delgado-Fukushima E.; Jihad T.; Liu C.-F.; O’Neill S.; Tu R. S.; Renfrew P. D.; et al. Thermoresponsive Protein-Engineered Coiled-Coil Hydrogel for Sustained Small Molecule Release. Biomacromolecules 2019, 20 (9), 3340–3351. 10.1021/acs.biomac.9b00107. 31356057

[ref18] Hume J.; Sun J.; Jacquet R.; Renfrew P. D.; Martin J. A.; Bonneau R.; Gilchrist M. L.; Montclare J. K. Engineered Coiled-Coil Protein Microfibers. Biomacromolecules 2014, 15 (10), 3503–3510. 10.1021/bm5004948. 24941228

[ref19] Meleties M.; Britton D.; Katyal P.; Lin B.; Martineau R. L.; Gupta M. K.; Montclare J. K. High-Throughput Microrheology for the Assessment of Protein Gelation Kinetics. Macromolecules 2022, 55 (4), 1239–1247. 10.1021/acs.macromol.1c02281.

[ref20] Meleties M.; Katyal P.; Lin B.; Britton D.; Montclare J. K. Self-Assembly of Stimuli-Responsive Coiled-Coil Hydrogels. Soft Matter 2021, 17, 6470–6476. 10.1039/D1SM00780G. 34137426

[ref21] Sharma N.; Furter R.; Kast P.; Tirrell D. A. Efficient introduction of aryl bromide functionality into proteins in vivo. FEBS Lett. 2000, 467 (1), 37–40. 10.1016/S0014-5793(00)01120-0. 10664452

[ref22] Pymol. The PyMOL Molecular Graphics System, Version 20; Shrödinger, LLC.

[ref23] Woolfson D. N. The design of coiled-coil structures and assemblies. Adv. Protein Chem. 2005, 70, 79–112. 10.1016/S0065-3233(05)70004-8. 15837514

[ref24] Britton D.; Almanzar D.; Xiao Y.; Shih H.-W.; Legocki J.; Rabbani P.; Montclare J. K. Exosome Loaded Protein Hydrogel for Enhanced Gelation Kinetics and Wound Healing. ACS Applied Bio Materials 2024, 7 (9), 5992–6000. 10.1021/acsabm.4c00569. PMC11409212 39173187

[ref25] Ewoldt R. H.; Johnston M. T.; Caretta L. M. Experimental Challenges of Shear Rheology: How to Avoid Bad Data. In Complex Fluids in Biological Systems: Experiment, Theory, and Computation, Spagnolie S. E., Ed.; Springer: New York, 2015; pp 207–241.

[ref26] Hill L.; Britton D.; Jihad T.; Punia K.; Xie X.; Delgado-Fukushima E.; Liu C. F.; Mishkit O.; Liu C.; Hu C. T.; et al. Engineered Protein-Iron Oxide Hybrid Biomaterial for MRI-traceable Drug Encapsulation. Molecular Systems Design & Engineering 2022, 7, 915 10.1039/D2ME00002D. 37274761 PMC10237276

[ref27] Schultz K. M.; Anseth K. S. Monitoring degradation of matrix metalloproteinases-cleavable PEG hydrogels via multiple particle tracking microrheology. Soft Matter 2013, 9 (5), 1570–1579. 10.1039/C2SM27303A.

[ref28] Kwok S. C.; Hodges R. S. Stabilizing and destabilizing clusters in the hydrophobic core of long two-stranded alpha-helical coiled-coils. J. Biol. Chem. 2004, 279 (20), 21576–21588. 10.1074/jbc.M401074200. 15020585

[ref29] Lau S. Y.; Taneja A. K.; Hodges R. S. Synthesis of a model protein of defined secondary and quaternary structure. Effect of chain length on the stabilization and formation of two-stranded alpha-helical coiled-coils. J. Biol. Chem. 1984, 259 (21), 13253–13261. 10.1016/S0021-9258(18)90686-1. 6490655

[ref30] Shepherd N. E.; Hoang H. N.; Abbenante G.; Fairlie D. P. Left- and right-handed alpha-helical turns in homo- and hetero-chiral helical scaffolds. J. Am. Chem. Soc. 2009, 131 (43), 15877–15886. 10.1021/ja9065283. 19807085

[ref31] Einstein A. Eine neue Bestimmung der Moleküldimensionen. Annalen der Physik 1906, 324 (2), 289–306. 10.1002/andp.19063240204.

[ref32] Korting H. C.; Braun-Falco O. The effect of detergents on skin pH and its consequences. Clinics in Dermatology 1996, 14 (1), 23–27. 10.1016/0738-081X(95)00104-N. 8901395

[ref33] Kwak M.-S.; Ahn H.-J.; Song K.-W. Rheological investigation of body cream and body lotion in actual application conditions. Korea-Australia Rheology Journal 2015, 27 (3), 241–251. 10.1007/s13367-015-0024-x.

[ref34] Andritoiu C. V.; Andriescu C. E.; Ibanescu C.; Lungu C.; Ivanescu B.; Vlase L.; Havarneanu C.; Popa M. Effects and Characterization of Some Topical Ointments Based on Vegetal Extracts on Incision, Excision, and Thermal Wound Models. Molecules (Basel, Switzerland) 2020, 25, E5356 10.3390/molecules25225356. PMC7709045 33207838

[ref35] Kamaruzaman N.; Yusop S. M. Determination of stability of cosmetic formulations incorporated with water-soluble elastin isolated from poultry. Journal of King Saud University - Science 2021, 33 (6), 101519 10.1016/j.jksus.2021.101519.

[ref36] Barnes T. M.; Mijaljica D.; Townley J. P.; Spada F.; Harrison I. P. Vehicles for Drug Delivery and Cosmetic Moisturizers: Review and Comparison. Pharmaceutics 2021, 13 (12), 2012 10.3390/pharmaceutics13122012. 34959294 PMC8703425

