# Supplementary material for: Recombinant Fibrous Protein Gels as Rheological Modifiers in Skin Ointments
Source: ACS Appl Polym Mater. 2024 Oct 8;6(20):12832–41. doi: 10.1021/acsapm.4c02468 (PMC11519833; doi:10.1021/acsapm.4c02468)
Supplement: Supplementary file 1 — ap4c02468_si_001.pdf [file ap4c02468_si_001.pdf]

Supplementary Information

Recombinant Fibrous Protein Gels as Rheological Modifiers in Skin Ointments

Dustin Britton[۱], *Jonathan Sun*[۱،۲], *Hammad Ali Faizi*[۴], *Ligeng Yin*[۵], *Wei Gao*[۶], *Jin Kim Montclare*[۱،۲،۷،۸،۹*]

[۱]Department of Chemical and Biomolecular Engineering, New York University Tandon School of Engineering, Brooklyn, New York, 11201, USA

[۲] Department of Chemistry, New York University, New York, New York, 10012, USA

[۴] The Dow Chemical Company, Home and Personal Care, Midland, Michigan 48611, USA

[۵] The Dow Chemical Company, Home and Personal Care, Collegeville, PA 19426, USA

[۶] The Dow Chemical Company, Analytical Science, Collegeville, PA 19426, USA

[۷] Bernard and Irene Schwartz Center for Biomedical Imaging, Department of Radiology, New York University School of Medicine, New York, New York, 10016, USA

[۸] Department of Biomaterials, New York University College of Dentistry, New York, New York, 10010, USA

[۹] Department of Biomedical Engineering, New York University, New York, NY, 11201, USA

* Corresponding author

Email: montclare@nyu.edu

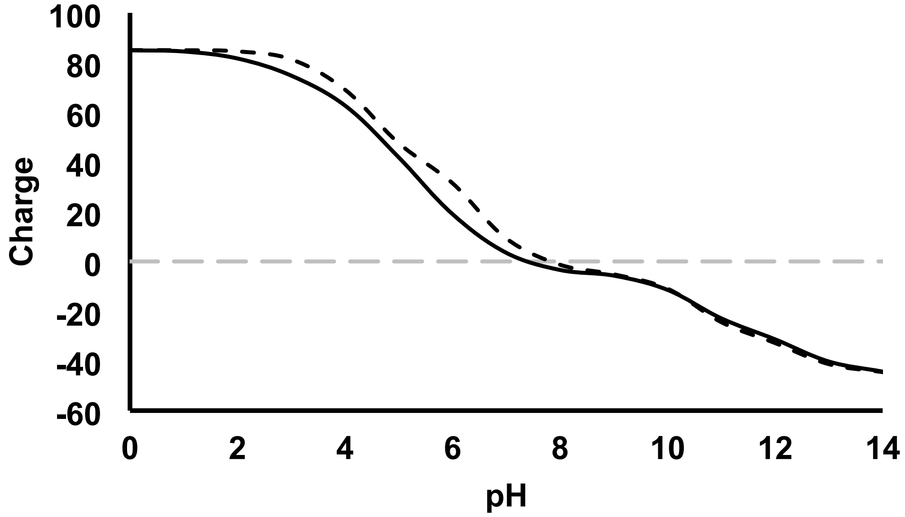

Figure S1 Calculated net charge using PDB2PQR of Q5 pdb model as a folded (solid line) and unfolded (dashed line) as a function of pH.

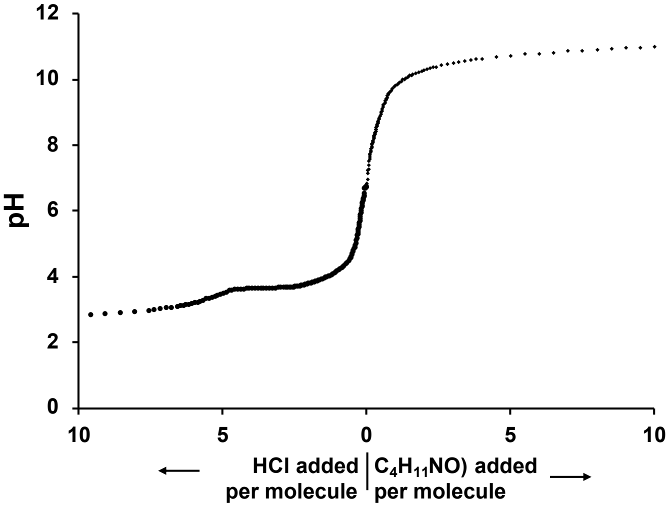

Figure S2 Titration curve of Q5 in buffer after addition of acid (HCl) or base ($C_4H_{11}NO$).

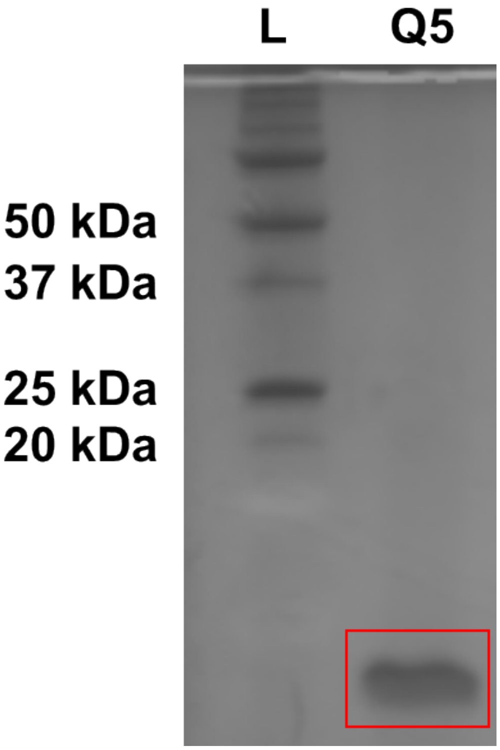

Figure S3 12% SDS-PAGE purity gel following purification and dialysis of Q5.

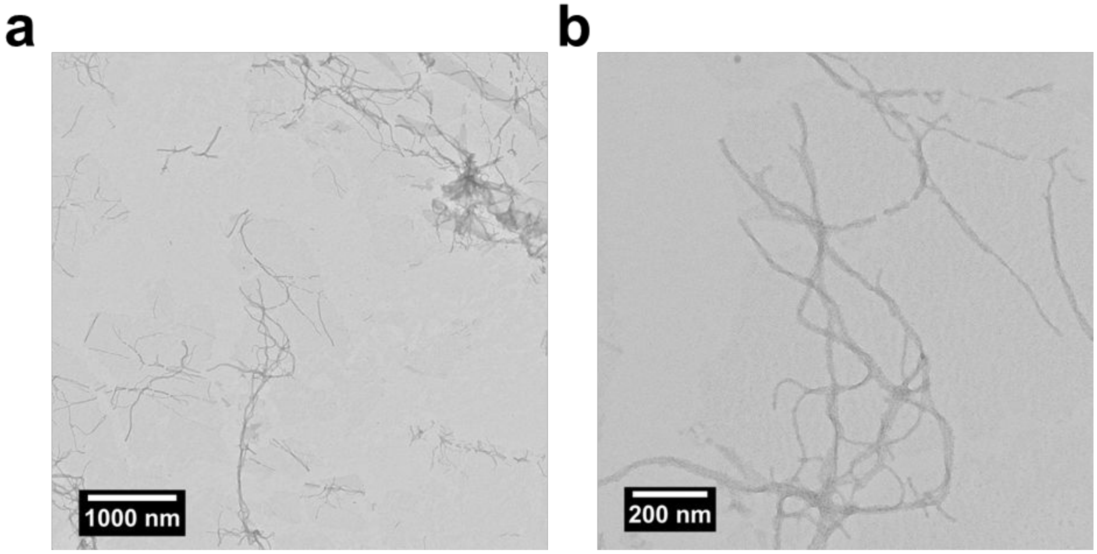

Figure S4 Transmission electron micrographs of Q5 at pH 8.0 showing isolation of physically crosslinked fiber groups at **a.** 11 kX and **b.** 45 kX resolution.

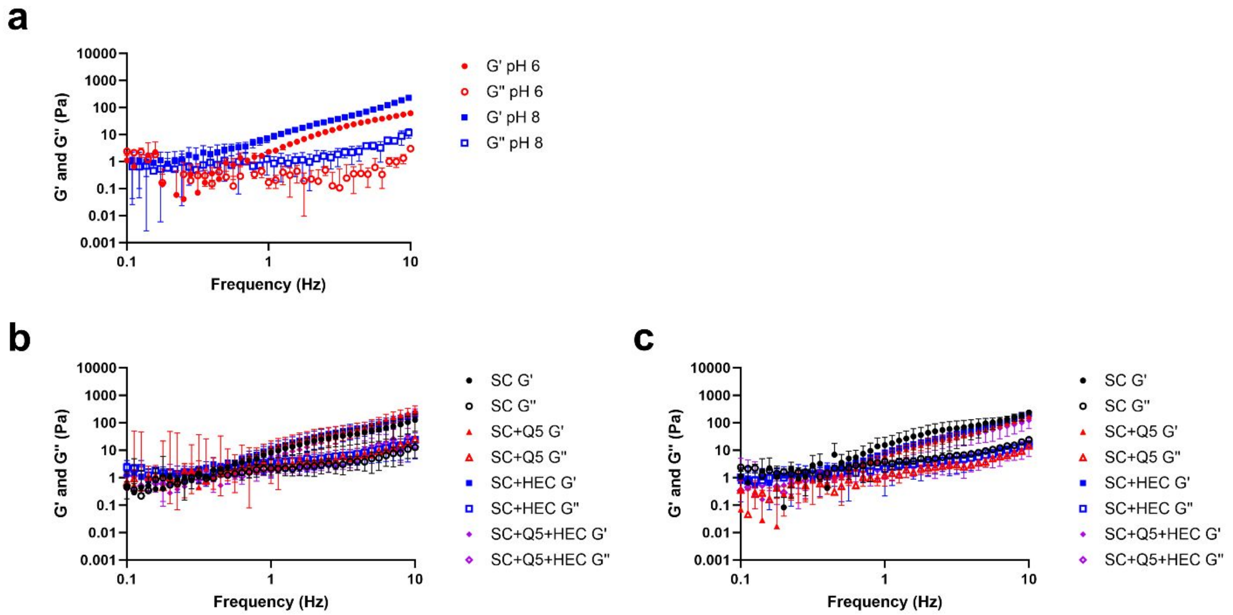

Figure S5 Rheology measured as a frequency sweep of **a.** Q5 at pH 6.0 (red) and pH 8.0 (blue) and SC formulations at **b.** pH 6.0 and **c.** pH 8.0 for average storage modulus (G’, filled markers) and average loss modulus (G’’, empty markers).

Table S1 CD compositional analysis of helical double minima and ATR-FTIR secondary structure deconvolution for Q5 at pH 6.0 and pH 8.0 in the solution (sol) and gel state. Average and standard deviations are from three independent trials. Data for CD measurements and ATR-FTIR of Q5 at pH 8.0 is reproduced from Britton *et al*, Biomacromolecules, 2024 licensed under CC BY 4.0.

	**Circular Dichroism** **Mean Residue Ellipticity (θ) minima ($\times 10^3$ deg cm^2 dmol^{-1})**			**ATR-FTIR** **% Secondary Structure**		
	**$-\theta_{208}$**	**$-\theta_{222}$**	**$\theta_{222}/\theta_{208}$**	**α-helix**	**β-sheet**	**Random Coil**
**pH 6.0 Sol**	12 ± 4	14 ± 3	1.3 ± 0.2	43.1 ± 6.9	30.0 ± 6.0	32.1 ± 11.5
**pH 6.0 Gel**	12 ± 1	11 ± 2	1.1 ± 0.1	20.2 ± 7.7	41.0 ± 7.4	29.3 ± 6.2
**pH 8.0 Sol**	16,000 ± 2,000	18,000 ± 1,000	1.2 ± 0.1	44.2 ± 2.9	39.8 ± 0.7	16.1 ± 3.3
**pH 8.0 Gel**	5,000 ± 1,000	7,000 ± 1,000	1.6 ± 0.1	48.6 ± 6.0	41.2 ± 3.8	10.0 ± 8.3

Table S2 Formulation composition for skin chassis with and without HEC.

	Ingredients %								
	**Phase A**			**Phase B**					**Phase C**
	Water	Cellosize PCG-10	Glycerin	Glyceryl Stearate, PEG-100, Stearate, Arlacel 165	Cetearyl Alochol	Stearyl Alochol	Coconut Oil	Caprylic/Capric Triglyceride, Crodamol GTCC	Phenoxyehtanol and Ethylhexylglycerin, Euxyl PE 9010
**SC**	80	0	2	1.5	0.8	0.8	4	11.25	0.5
**SC+HEC**	79	0.2	2	1.5	0.8	0.8	4	11.25	0.5
